# Stromal circuits involving tumor-associated macrophages and cancer-associated fibroblasts

**DOI:** 10.3389/fimmu.2023.1194642

**Published:** 2023-06-05

**Authors:** Eleonora Timperi, Emanuela Romano

**Affiliations:** ^1^ Department of Immunology, INSERM U932, Université Paris Sciences et Lettres (PSL) Research University, Institut Curie, Paris, France; ^2^ Department of Medical Oncology, Center for Cancer Immunotherapy, Institut Curie, Paris, France

**Keywords:** cancer associated fibroblasts (CAF), tumor associated macrophages (TAM), solid tumors, single cell RNA analysis, monocytes

## Abstract

The tumor associated macrophages (TAM) represent one of most abundant subpopulations across several solid cancers and their number/frequency is associated with a poor clinical outcome. It has been clearly demonstrated that stromal cells, such as the cancer associated fibroblasts (CAFs), may orchestrate TAM recruitment, survival and reprogramming. Today, single cell-RNA sequencing (sc-RNA seq) technologies allowed a more granular knowledge about TAMs and CAFs phenotypical and functional programs. In this mini-review we discuss the recent discoveries in the sc-RNA seq field focusing on TAM and CAF identity and their crosstalk in the tumor microenvironment (TME) of solid cancers.

## 1 Introduction

The advent of sc-technologies has fast-revolutionized our understanding about macrophage phenotype, function, and plasticity in several diseases, including cancer. The binary view of macrophage states: M1 and M2, has dominated the field until recently. M1 (pro-inflammatory) versus M2 (alternative or anti-inflammatory) profiles were derived by *in vitro* observations in human and mice (1). M1- macrophages, obtained *in vitro* by type 1 cytokines such as IFN-γ (and/or TNF-α) showed efficient phagocytosis, high levels of pro-inflammatory cytokines (i.e. IFN-γ, IL-12, TNF-α) and chemokines (i.e. CCL2, CXCL10). Conversely, the generation of M2-macrophages, was mainly induced by type 2 cytokines like IL-4 and/or IL-13 (1). M2-like macrophages are characterized by increased wound healing activity, reduced phagocytosis and T cell antigen presentation capacity (2, 3). Recent sc- discoveries revealed that human macrophages are highly heterogeneous at the steady state and in pathological conditions, suggesting the importance of a context- and tissue-dependent approach to appreciate their biological properties.

## 2 TAM: tissue resident macrophages and monocyte-derived TAM in tumor niches

TAM are one of the most abundant population in solid cancers (4). TAM density is linked to poor patient outcome in prostate (PCA), breast (BC), bladder, head and neck (HN), glioma, melanoma, thyroid, lung (NSCLC), hepatocellular (HCC) cancers, and non-Hodgkin lymphoma (5–10). Collectively, TAM may originate from tissue-resident macrophages (TRM) and circulating monocytes (mono)-derived cells. This review will describe recent discoveries on the aspects linked to the TAM origin.

All the organs in the body are populated by (TRM), key players in mounting the first-line of defense against pathogens, preserving vascular tone and integrity, in addition to clearance foreign bodies (11). Embryonically generated-TRM preserve the organ homeostasis at steady state. In response to inflammation, TRM may be originated by circulating monocytes. The contribution by peripheral monocytes could be driven by the inability of TRM to generate macrophages with specific effector functions in the tissue, because of the limited TRM self-renewal intrinsic capacity (11). Upon infections or inflammation, bone-marrow- adult derived cells could be recruited at the tissue and replaced embryonic-TRM. Among many inflammatory triggers (i.e. infections), cancer-associated inflammation may be considered a key perturbator of the frequency of TRM across multiple cancer subtypes. Indeed, circulating monocytes may be recruited by the engagement of various chemoattractant pathway by the interplay of stromal components like CCL2-CCR2, CCL20-CCR6, CCL5-CCR5, CCL8- CXCR4-CXCL12 etc (12). At the tumor site, monocytes undergo gene reprogramming and acquire similar properties of embryonically originated macrophages, depending on specific tissue factors (2, 13–15). Chronic inflammation of different etiology can give rise to the differentiation of recently recruited monocytes towards TAM at the tissue site.

So far, sc-RNA seq technologies have contributed to defining i) the theoretical origin of TAM; ii) TAM heterogeneity; iii) TAM molecular features iv) TAM functional and metabolic states. This large effort has contributed to understand which molecular programs are conserved among cancer types and which programs could be tumor tissue-specific.

## 3 TAM in the era of single cell RNA-sequencing technology

Most of the sc-datasets showed the APOE (apolipoprotein) gene as a TAM marker. Numerous studies, including our, demonstrated the selective APOE expression by TAM from tumor lesions compared with macrophages from normal-tissue (NT) counterparts (16–18). Despite tissue resident (TR) or monocytic origin of TAM, they may collectively share a core transcriptomic signature comprising: APOE, complement component genes (i.e. C1QA, C1QB, C1QC), and cathepsin (CTSB, CTSD) across several cancer types (16, 18–21).

### 3.1 TR-derived TAM

TAM derived from TRM were described in several cancer tissues. In human colorectal cancer (CRC), C1QC+ TR-TAMs were identified, showing high complement components (C1QA, C1QC etc.), high levels of HLA-DR molecules and high phagocytic score (20). Importantly, Cheng et al, collecting sc-RNA data from 15 different cancer subtypes, reported that C1QC+ TAM showed a lower connectivity with CD14+ monocytes suggesting their TR origin (19). Of note, the folate receptor-β (FOLR2) has been recently discovered and described as TR marker. In HCC FOLR2+ TAM exhibited fetal-liver features and displayed onco-fetal reprogramming (22), supporting their resident origin. TR FOLR2+ macrophages have been also identified (16) in breast cancer (BC) lesions and in healthy mammary tissues; they were associated with high CD8+ T cell infiltration and better prognosis. Additionally, mannose receptor C, type 1 (MRC1) and perivascular markers like Lymphatic Vessel Endothelial Hyaluronan Receptor 1 (LYVE1) and stabilin-1 (STAB1) were expressed by the FOLR2+ TR-TAMs. In agreement with the expression of perivascular markers, fetal-derived mammary gland macrophages display periductal and perivascular localization (23). In accordance, Cheng et al, demonstrated highest similarities between LVYE1+ TRM and FOLR2+ TR-TAMs. Since LYVE1+ macrophages were identified in multiple cancers and preferentially enriched in NT counterpart (19), the authors suggested that the enrichment of LVYE1+ TRM in adjacent NT may function as the potential pool for the FOLR2+ TAMs. Many observations suggested therefore a protective role for TRM in some cancers, however, other findings proposed that in lung and pancreas lesions, TRM played a key role in tumor initiation (24, 25). In non-small lung cancer TR alveolar TAM may induce epithelial-mesenchymal transition (EMT), regulatory T cell activation and promoting pro-tumorigenic fibroblast-TRM crosstalk, finally fostering tumor progression and invasiveness (25).

### 3.2 Mono-derived TAM

Tumor-infiltrating mono-derived TAM were described in a variety of human and murine cancer models. Müller and collaborators have been pioneers in dissecting the transcriptomic properties of mono-derived TAMs in gliomas. They demonstrated the co-existence of CX3C motif chemokine receptor 1 (CX3CR1)- blood-derived TAM, CX3CR1+ blood-derived TAM and lastly CD11b+CX3CR1+HLADRlow as TR microglia (26). Corroborating studies by Friebel and collaborators have defined TAM heterogeneity in primary gliomas and brain-derived metastasis. They demonstrated a mono-derived TAM cluster expressing CD163, CD206 and one expressing high level of Cell Adhesion Molecule 1 (CADM1) and CX3CR1 (27). In line, a study in BC identified CADM1 as marker of mono-derived TAM (16). Collectively, all these studies proposed CX3CR1 and CADM1 as mono-derived TAM markers (28).

The lipid-associated TREM2 (Triggering Receptor Expressed on Myeloid Cells 2) receptor has been recently associated to mono-derived TAM in many cancer subtypes (17, 28–32). Its expression was detected together with APOE, APOC1 (apolipoprotein C1), FABP5 (fatty acid binding protein) and LIPA (Lipase A), genes involved in lipid transport and metabolism and highly detected in breast, sarcoma, colon, lung and other cancers (17, 28–31). Our work and that of others (17, 33) suggested that TREM2+ TAMs bear close transcriptomic profile to a Lipid Associated Macrophage (LAM) subpopulation, highly enriched in the adipose tissue of obese patients and in mice fed with high fat diet (34). These LAM were described as mono-derived cells (17, 33). Lipid-associated molecular profiles were highly enriched in several tumors and associated with a detrimental role in cancer progression. For example, Masetti et al, have demonstrated that MARCO+ TAM expressed high lipid-content and lipid-associated molecular signatures in prostate cancer, similarly lipid-laden TAMs have been discovered by Di Conza et al. (35, 36). Lipid loaded TAM or/and LAM were associated with poor prognosis and outcome (17, 33, 35, 36) suggesting a protumor role for lipids. Intriguingly, several groups have demonstrated that the abrogation of TREM2 activity in mice, by *Trem2* KO models or by Trem2 antibody-based blocked therapies, induced tumor growth delay and synergistic effect on T cell restoration functions concomitantly with anti-PD-1 blockade in many mouse models (CRC, sarcoma) (28). Although the mechanism of Trem2-/- KO or blockade activity seems to be T cell dependent, it remains to be elucidated the blocking effect of Trem2 as lipid marker in cancers. Overall these studies demonstrated a pro-tumoral role for mono-derived TREM2 TAM.

**Figure 1:**
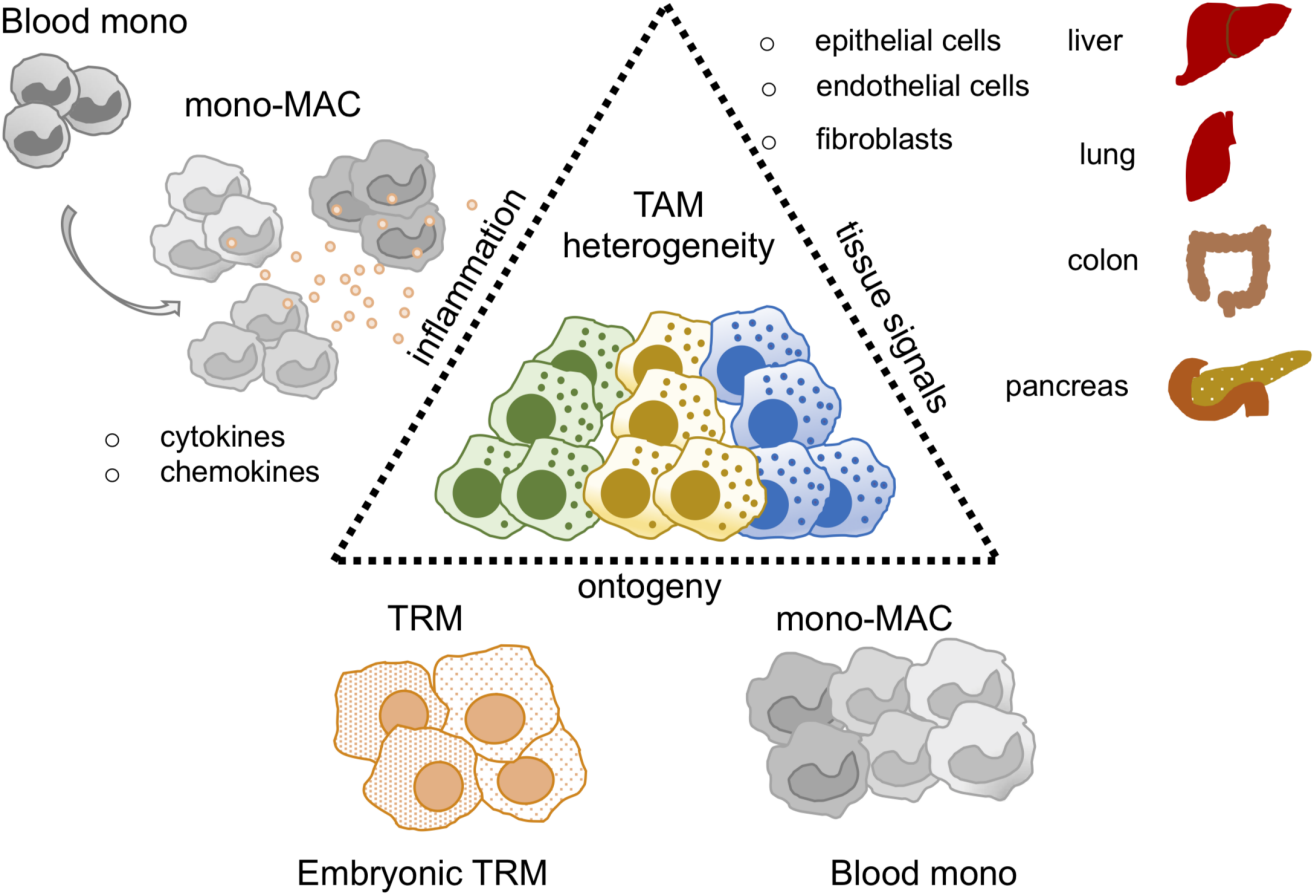
TAM heterogeneity in the sc-RNAseq era. Thanks to sc-RNAseq studies TAM heterogeneity has been revised. Key factors described to shape the TAM identity are: i) tissue signals, mediated by epithelial, endothelial and fibroblast cells represented in each organ of interest, ii) ontogeny, TAM may derive from tissue resident macrophages (TRM) or blood monocytes (Blood mono), iii) inflammation, it may influence and balance the recruitment of blood mono at tumor site perturbing TRM/blood monocyte ratio in the tumor.

Another mono-derived marker commonly identified is the SPP1 (Osteopontin) gene (37). Of note, Zhang and colleagues demonstrated that a subset of SPP1+ TAMs may be developed from tumor-infiltrating mono-like precursors in CRC lesions (20). SPP1+ TAMs were described in 8 cancer subtypes: BC, PCA, Lung, CRC, Uterine corpus endometrial, Nasopharyngeal, Ovarian and Thyroid carcinoma, preferentially expressing an angiogenic signature (19). Some of them expressed high levels of MARCO gene, and Zhang et al, demonstrated that IL-1β and VEGF were able to upregulate its expression under hypoxic conditions (20). Collectively, SPP1 mono-derived TAM were associated with protumor and M2-like signatures, proposing a protumor role for these cells. Conversely to the observations above, mono-derived SPP1+ TAM have been recently identified associated to protective CXCL13+ T cell responses and highly correlated with plasma B cells, indicating a protective SPP1+ TAM role in human lung cancer (30). The large contribution of sc-datasets in identifying several TAM clusters highlighted the importance of having a consensus annotation. A big effort has been done by Mulder et al, in providing a robust online-available platform with the aim to harmonize the annotations of macrophages in healthy and pathological states. The authors have generated a monocyte-macrophage compendium widely distributed across multiple tissues. Some TAMs were exclusively expanded in cancer and inflamed tissues and generally enriched in neoplastic lesions (37). In pursuing the effort of collecting shared TAM features, Cheng and colleagues have demonstrated that - in a large cohort of 15 different cancer subtypes - TAM subsets could be concomitantly identified across cancer subtypes. However, the similarity analysis failed to exactly cluster TAMs with the same identity. These observations indicated that TAM exhibited high levels of complexity and heterogeneity, highlighting the crucial role for the local tissue microenvironment in shaping the TAM phenotype (14, 22) (**Figure 1**).

## 4 Introduction to CAFs

The TME is a complex ecosystem where the malignant cells coexist with immune and stromal cells (fibroblasts and endothelial cells). CAFs represent the predominant cell type. CAFs play tumorigenic roles by promoting cancer cell survival and proliferation, inducing angiogenesis and extracellular matrix (ECM) remodeling. CAF subsets have been described to modulate immune responses, inducing regulatory T cell programs, T cell suppressive activities and recruiting myeloid cells at the tumor site (38). The peptidase inhibitor 16 (PI16) gene is considered a universal fibroblast marker, mostly expressed by normal fibroblasts (NFs) from NT areas (39). Conversely, CAFs expressed specific markers, less or not expressed by NFs, such as alfa-SMA (a-SMA), fibroblast activated protein (FAP), fibroblast specific protein-1 (FSP1), platelet derived growth factor receptor (PDGFR-α-β and podoplanin (PDPN) (40–44).

### 4.1 CAF in the era of single cell RNA-sequencing technology

FAP+ CAFs showed an activated phenotype compared to NFs and they were strongly enriched in tumor lesions compared with NT (38). Activated FAP+ CAF expressed pathways involved in collagen activation, ECM, metalloproteinase-related genes, adhesion and wound-healing signatures (45).

Thanks to the sc-RNA studies FAP+ CAFs have been deeply phenotyping, and different groups have observed highly heterogeneity of this subpopulation in NSCLC (30, 46–49), bladder (50), pancreas (51, 52), BC (53), liver (54) and HN (55) tumors.

Öhlund and colleagues have described that FAP^high^ CAFs comprised matrix-producing myo-fibroblastic phenotype (myCAF) and immunomodulatory secretome or inflammatory CAFs (iCAF) in human PCA and pancreatic mouse model. iCAF were able to produce high levels of IL-6, IL-11, leukemia inhibitory factor (LIF), and chemokines (CXCL1, CXCL2) while myCAF, detected closer to the tumor lesions, expressed high levels of α-SMA and ACTA2 genes, CTGF and COL1A1 (TGF-β-response genes) (51). Kieffer at al., have corroborated these observations in BC, distinguishing ANTRX1+ myCAF from ANTXR1- iCAFs. myCAF comprised ecm-myCAF, TGFβ-myCAF, and wound-myCAF involved respectively in extracellular matrix organization pathway, TGF-β pathway, collagen fibril organization and wound healing pathway. Whilst iCAF included subsets deputized to cytokine/chemokines production: detoxCAF (closer to NFs phenotype), IL-iCAF (deputized to cytokine/chemokines productions) and IFN-iCAF (involved in cytokine-mediated response to interferon-gamma genes) (53). The authors demonstrated that myCAFs correlated with non-responder patients to immune checkpoint blockade (ICB) therapies, demonstrating a role of FAP+ CAF in contributing to primary resistance to immunotherapy. Another study demonstrated the presence of leucine-rich-repeat-containing protein 15 (LRRC15+) myCAFs able to directly suppress CD8 T cell function and limit responsiveness to ICB (52). myCAF and iCAF subsets were accordingly identified in triple negative breast cancer (TNBC) and CRC (56, 57). Generally, these data suggested iCAF distal from the tumor lesion and with secretory ability, while myCAF, described in close proximity to the tumor site, showed activated and contractility genes (51, 56, 57). Of interest, Grout et al, dissected NSCLC stromal TME. They identified alcohol dehydrogenase 1B (ADH1B) positive CAFs, carrying low activation state and highly producing CCL19, they were spread throughout the stroma and supported a T-cell permissive TME. In contrast, MYH11+αSMA+ CAFs expressing myosin heavy chain 11 (MYH11) gene, ACTA2, and intermediate levels of CD34 were localized as a single layer encapsulating the tumor nest and orchestrating T-cell exclusion. Both ADH1B+ and MYH11+αSMA+ and CAFs characterized early stage of the disease. At advanced stages other two clusters were identified: FAP+ CAFs expressed high levels of periostin (POSTN), Leucine Rich Repeat Containing 15 (LRRC15), and Gremlin1 (GREM1) genes and FAP+ αSMA+ CAFs. Intriguingly, while FAP+ αSMA+ orchestrated T-cell exclusion, FAP+ CAF showed T-cell permissive TME (47). This study has elucidated the importance of different CAF subpopulations at displaying T-cell permissive or excluding TME. Still remains to understand which factors influence CAF subtypes. Of remarkable interest for the immunologists was the discovery of antigen-presenting CAFs (apCAFs) in mouse and human PCA ductal adenocarcinoma. Elyada et al, showed that apCAFs expressed high levels of MHC-class II genes (H2-Ab1) and CD74 gene, however they did not express classic costimulatory molecules. They expressed markers regulating the immune system like BCAM (CD239), F11R (member of Immunoglobulin genes), IRF5 (interferon stimulating factor 5) and STAT1, known to mediate MHC expression in response to IFN-γ. These MHC class II–expressing CAFs showed the capacity to activate CD4+ T cells in an antigen-specific manner, corroborating their putative immune-modulatory aptitude (58). Rapidly, our view about CAFs and their heterogeneity has changed. The coexistence of myCAF and iCAF in the TME suggests a compartmentalization, both in terms of localization (close or distant to the tumor nest) and functions that may dictate the localization and the phenotype/function of tumor-infiltrating immune cells. Due to the availability of numerous sc-RNA seq datasets and given the deep-phenotyping of CAFs and TAM in many cancer studies, CAF-TAM interactions and their cross-talks has been reviewed.

### 4.2 CAF and TAM crosstalk in the TME

At steady-state the connection between fibroblasts and macrophages is documented by the ability of NFs to produce colony stimulating growth factor 1 (CSF-1), lineage-specific growth factor, crucial for the proliferation and survival of macrophages. Zhou et al., have demonstrated that microenvironmental sensing by fibroblasts may control macrophage population size by producing CSF-1 (59). CAFs and TAMs may interact via the CSF1-CSF1R axis also in the TME (60). So far, it has been collectively demonstrated that CAFs may secrete several factors well-known to influence the recruitment and activation state of myeloid cells including: IL-1β, IL-8, IL-6, IL-33, IL-10, Chi3L1, CXCL1, CXCL2, CXCL5, CXCL6, CXCL8, CXCL9, CXCL10, CXCL16, CXCL12/SDF1, CCL2/MCP-1, CCL3, CCL5/Rantes, CCL7, CCL20, CCL26, TGF-β, prostaglandin (PGE2), indoleamine-2,3-dioxygenase (IDO), LIF, VEGF, tumor necrosis factor (TNF), and nitric oxide (NO) (61–63). CAFs may recruit monocyte at the tumor site by CCL2-CCR2 pathway. FAP+ CAFs were identified as a major source of CCL2 in intrahepatic cholangiocarcinoma (64). The CCL2-CCR2 axis was also linked to tumor progression in a spontaneous model of lymphoma; accordingly, genetic ablation of Ccr2 inhibited tumor growth (65). CAFs may promote skin carcinogenesis by maintaining CCL2 mediated monocyte/macrophage infiltration and chronic inflammation (66). CAF derived-CXCL16 chemokine may also recruit mono promoting stromal activation and then tumor progression in TNBC (67). CXCL14 may be produced by CAFs, therefore amplifying mono recruitment at tumor site and acting as stimulator of prostate tumor growth (68). Among the pathways involved in the mono recruitment, CXCL12 is well studied. CAFs produce high levels of CXCL12 in the TME and CXCL12-CXCR4 CAF-TAM axis is responsible for mono recruitment at the tissue (69). In line, targeting the CXCL12 pathway from FAP+ CAFs synergized with anti–PD-L1 immunotherapy in PCA (70). In bladder cancer CXCL12-CXCR4 iCAF-TAM crosstalk was described (50). Our study, in accordance with other studies, demonstrated that iCAF, highly enriched in TNBC, were the major source of CXCL12, resulting the key cells sustaining the recruitment of CXCR4+ monocyte in TNBC (17). In keeping with our observations in TNBC, Wu and colleagues demonstrated that iCAF-TAM crosstalk strongly associated with cytotoxic T-lymphocyte dysfunction in TNBC (57). Overall, the recruitment of monocytes via the CXCL12-CXCR4 axis was associated with tumor progression. iCAF-TAM axis mainly involved the complement cascade activation pathway by the interactions of complement C5-C5AR1. C5 pathway is an important chemotactic factor for the recruitment of immunosuppressive myeloid cells ultimately suppressing T-cell activities (71). A cross-talk between C3-C3aR iCAF-TAM axis has been additionally elucidated in melanoma, HN and BC (60). These data suggested that CD34+ PDPN+ and PDGFR-α+ iCAFs were highly producers of C3, C2, and C4b complement components, additionally to CXCL12, CSF-1 and CCL8 factors. CD34+ CAFs, by producing C3 and by the C3a conversion into an activated form in the TME, allowed the recruitment of C3aR+ circulating monocytes. By confocal microscopy analysis, C3aR+ TAMs were proximally located to CD34+ CAFs, indicative of a generation of supportive protumor niche by iCAF-TAM interactions (60). Globally these data suggested a pro-tumoral role for the complement components in recruiting circulating monocytes and favor immune suppression. These data supported a recent hypothesis that iCAF, rather than myCAF, may play a fundamental role in promoting tumor progression by recruiting monocytes at the tumor site via local inflammation. Among the pathways responsible of CAF monocyte reprogramming IL-6/STAT3 is well studied. CAF-derived IL6 leads to myeloid immunosuppression phenotype by STAT3 activation. Inhibiting IL-6 pathway or STAT3 activation by blocking CAF-TAM interactions decreased immunosuppression in PCA (72) and HCC (73) was observed. STAT3 activation is also mediated by LIF and IL-11. LIF pathway leaded to immunosuppressive signature on TAMs by decreasing CXCL9 expression and preventing cytotoxic CD8+ T-cell recruitment, impairing anti-PD1 response (74). In a model of BC CAF-derived Chi3L1 induced mono recruitment and M2-like TAM reprogramming by inducing CD206 and ARG1 expression.

Cytokines as IL-8, IL-33, IL-10, TGF-β and CCL2 secreted by CAFs promoted the recruitment of monocytes at tumor site and the M2-like protumor phenotype (66, 75, 76). Collectively, many studies have demonstrated CAF-mediated mechanisms inducing M2-like TAM phenotype (17, 77–80). Of note, Mazur et al., explained the mechanisms by which the FAP protein could interact with TAM. The authors have demonstrated that FAP is crucial for the CAF interaction with class A scavenger receptor (SR-A or CD204) expressed by TAM, mainly by cleaving type I collagen resulting in increased TAM adhesion (81). A protumor niche generated by the interactions between FAP+ CAF and SPP1+ TAM has been identified in CRC. The abundance of both FAP+ CAFs and SPP1+ TAMs was correlated with worst patient survival. Interestingly, FAP+ CAFs and SPP1+ TAMs were found in close proximity in the TME communicating by TGFβ-ACVRL1/ACVR1/B pathway, CCL3-CCR5 axis and RARRES2-CMKLR1 pathway. The latter involved in the recruitment of CMKLR1+ monocyte/TAM at the tumor site. These were described as pro-tumoral pathways in the tumor promotion and progression.

**Figure 2:**
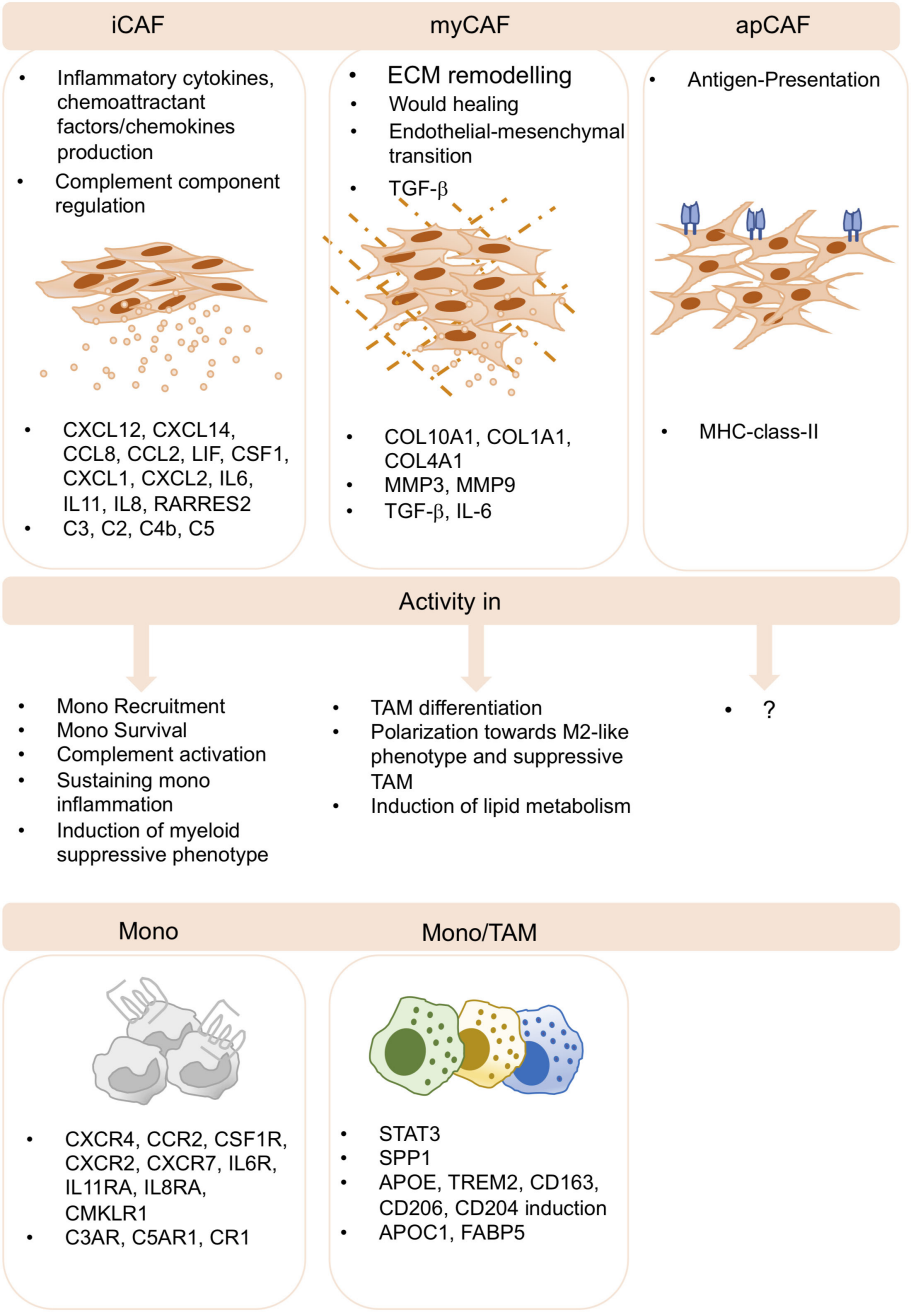
CAF-TAM interactions in the TME. Inflammatory CAF (iCAF), myofibroblasts CAF (myCAF) and antigen presenting CAF (apCAF) have been described by several sc-studies and across cancer subtypes. iCAF produces inflammatory cytokines and chemokines and they produce complement components. They play key roles in monocyte recruitment, inflammation, complement activation and in the induction of suppressive functions of myeloid cells. myCAF are involved in extracellular matrix remodeling, wound healing, endothelial-to-mesenchymal transition, and produce TGF-b. They induce M2-like phenotype, differentiation and polarization of suppressive TAM and the induction of lipid metabolism. apCAF have been described, however, no specific functions associated to TAM biology have been reported to date.

Since both FAP+ CAFs and SPP1+ TAMs were enriched in genes linked to ECM the authors suggested that this myCAF-TAM axis may facilitate the generation of desmoplastic structures in CRC (82). In agreement, a positive correlation between FAP+ CAF and SPP1+ mono-derived TAM was found in NSCLC cohort (47). Our study in TNBC demonstrated also a protumor niche between FAP+ CAF and mono-derived LAM. We have demonstrated by *in vitro* assays that FAP+ CAF were able to induce a LAM-like suppressive phenotype characterized by the induction of APOE, APOC1, FABP5, ACP5 and TREM2 genes. LAM-differentiated cells were able to inhibit T cell proliferation and activation state orchestrating suppressive functions (17). In keeping with these studies, a work collecting 10 cancer subtypes has demonstrated the existence of CAFs generated from endothelial cells by endothelial-mesenchymal transition (EndMT) (CAF-EndMT). They exhibited concomitant expression of CD44+CD31+ and ACTA2, in addition to regulator of G Protein Signaling 5 (RGS5), plasmalemmal vesicle-associated protein (PLVAP) and von willebrand factor (VWF) genes. The authors identified CD44+ CAF EndMT - Spp1+ TAM interactions in promoting EndMT process and angiogenesis leading to poor prognosis in cancer patients (45) (**Figure 2**).

## 5 Discussion

This review gathers evidence from key studies that highlight the suppressive crosstalk between newly identified TAM and CAF subpopulations across different solid cancers and explores the suppressive modules that could provide potential targets of new therapeutic approaches.

## Author contributions

ET and ER wrote and conceptualized the review, ET and ER reviewed the literature on single cell studies on tumor-associated macrophages and cancer associated fibroblasts. All authors contributed to the article and approved the submitted version.
